# Nanomedicines for the Efficient Treatment of Intracellular Bacteria: The “ART” Principle

**DOI:** 10.3389/fchem.2021.775682

**Published:** 2021-10-20

**Authors:** Hongzhao Qi, Peipei Shan, Yin Wang, Peifeng Li, Kun Wang, Lijun Yang

**Affiliations:** ^1^ Department of Aging Research, Institute of Translational Medicine, The Affiliated Hospital of Qingdao University, College of Medicine, Qingdao University, Qingdao, China; ^2^ Qingdao Institute of Bioenergy and Bioprocess Technology, Chinese Academy of Sciences, Qingdao, China

**Keywords:** intracellular bacteria, nanomedicines, accumulation, recognition, targeting

## Abstract

Infections induced by bacteria at present are a severe threat to public health. Compared with extracellular bacteria, intracellular bacteria are harder to get rid of and readily induce chronic inflammation as well as autoimmune disorders. As the development of new antibiotics becomes more and more difficult, the construction of new antibiotic dosage forms is one of the optimal choices for the elimination of intracellular bacteria, and, to date, various nanomedicines have been exploited. However, current nanomedicines have limited treatment efficiency for intracellular bacteria due to the multiple biological barriers. Here in this short review, we focus on systemically administered nanomedicines and divide the treatment of intracellular bacteria with nanomedicines into three steps: 1) Accumulation at the infection site; 2) Recognition of infected cells; 3) Targeting of intracellular bacteria. Furthermore, we summarize how nanomedicines are elaborately designed to achieve the "ART" principle and discuss the problems of experimental models construction. Through this review, we want to remind that the golden approach for the building of cell and animal experimental models should be established, and nanomedicines should be also endowed with the versatility to follow the “ART” principle, efficiently improving the treatment efficiency of nanomedicines for intracellular bacteria.

## Introduction

Infections induced by intracellular bacteria pose a formidable challenge to clinical therapy (Kamaruzzaman et al., 2017). These intracellular bacteria can invade into cells and survive in active or static states over extended periods. Under the appropriate conditions, they can quickly replicate inside cells resulting in the relapse of infections. Besides, intracellular bacteria can cause chronic inflammation as well as autoimmune disorders (Kaufmann, 2011). Numerous antibiotics, to date, have been clinically used to treat intracellular bacteria-induced infections, but it is hard to completely eradicate intracellular bacteria (Imbuluzqueta et al., 2011). On the one hand, parts of antibiotics such as penicillin and streptomycin possess high hydrophilicity and have restricted cellular penetration. On the other hand, although some antibiotics can readily diffuse into cells, they show low intracellular retention efficiency (Abed and Couvreur, 2014). Although the application of excessive doses of antibiotics can potentially enhance its intracellular concentration, various side effects and toxicities are often encountered (Qiu et al., 2017). In addition, the activity of antibiotics may also be influenced by intracellular factors such as pH, redox status, and enzymes (Wright, 2005). Taken together, the effective intracellular concentration of antibiotics is often subtherapeutic, resulting in low efficiency against intracellular bacteria and the development of antibiotic resistance.

To enhance the therapy efficiency of antibiotics for intracellular bacteria as well as avoid potential side effects and toxicities, different nanomedicines that can maintain the activity of drugs have been exploited (Qi et al., 2020a). A variety of drug delivery vehicles, such as polymeric nanoparticles (Anversa Dimer et al., 2020) and gold nanoparticles (Chowdhury et al., 2017), have been used to load and deliver antibiotics to kill intracellular bacteria, while the treatment efficiency of current nanomedicines is rather low. In previous researches, we have developed a series of nanomedicines for the treatment of various diseases (Qi et al., 2016; Qi et al., 2018; Han et al., 2019; Qi et al., 2019; Qi et al., 2020b). Therefore, based on our experience, the optimal nanomedicine should undertake the following three steps for the efficient treatment of intracellular bacteria: 1) Accumulation at the infection site; 2) Recognition of infected cells; 3) Targeting of intracellular bacteria. Nanomedicines that follow this “ART” principle can work like guided missiles to precisely eradicate intracellular bacteria. In this mini-review, we will summarize how nanomedicines are elaborately designed to achieve the “ART” principle, facilitating the exploitation of novel nanomedicines for the efficient treatment of intracellular bacteria. It should be noted that we just discuss nanomedicines which are systemically administered in this mini-review because different administration ways may need nanomedicines with different properties.

## Accumulation at the Site of Infection

Intracellular bacteria can induce local infections such as skin infections and lung infections (Shima et al., 2016). For the treatment of these infections, nanomedicines have to be accumulated at the site of these infections firstly. The targeting mechanisms of nanomedicines include passive targeting and active targeting. Passive targeting depends on the physicochemical properties of nanomedicines, while active targeting mainly relies on the modification of targeting moieties. Below we will discuss these two aspects in great detail.

### Passive Targeting of Nanomedicines

It has been reported that capillaries in infection sites are often damaged, and the capillary permeability is positively correlated with the severity of infection (Schiffelers et al., 2001). This physiological characteristic plays a crucial role in nanomedicines localization at the infection sites. Owing to the microvascular permeability, nanomedicines can passively extravasate from blood vessels to the infection sites. In the passive targeting process, besides the physiological characteristics of infection sites, physicochemical properties of nanomedicines such as sizes and zeta potentials also affect the targeting efficiency. For example, Fenaroli et al. have reported that PEGylated liposomes (190 nm) accumulate at infection sites more than PEGylated ones (101 nm), while PEGylated liposomes (703 nm) have a lower accumulation efficiency (Fenaroli et al., 2018). They also prove that PEGylated liposomes (101 nm) have a much longer blood circulation time than non-PEGylated liposomes (101 nm), and their accumulation efficiency at infection sites is significantly higher. Furthermore, to enhance the accumulation efficiency, nanomedicines are often prepared with neutral or negative surface charge, potentially reducing the opsonization and avoiding the quick clearance by the immune system (Xiao et al., 2011). In conclusion, nanomedicines can be endowed with long circulation characteristics by controlling their size and surface properties, and these nanomedicines have higher passive targeting efficiency to infection sites.

It should be noted that, however, nanomedicines that are readily captured by the immune system can potentially show excellent targeting efficiency to infection sites. As an example, rifampicin-loaded PLGA nanoparticles can be taken up by macrophages, and these macrophages would carry nanoparticles to the infection sites because of their chemotaxis (Fenaroli et al., 2014). Even so, this indirectly passive targeting of nanomedicines is potentially inefficient because macrophages may result in the degradation and inactivation of nanomedicines. The application potentials of indirectly passive targeting need further verification.

Passive targeting of nanomedicines to infection sites is based on the enhanced permeability and retention (EPR) effect similar to that of nanomedicines to tumors (Yang et al., 2019). The vasculatures at the tumor sites and the infection sites are similar, and they are often leaky and have high permeability. But the formation mechanism of the EPR effect may be different because the microenvironment of tumor and infection is different. Despite this, the heterogeneity of the EPR effect both at the tumor sites and infection sites has been widely discussed, and it can reduce the passive targeting efficiency (Fang et al., 2020). To reduce the influence of vascular heterogeneity, nanomedicines with active targeting ability have been exploited.

### Active Targeting of Nanomedicines

Nanomedicines with active targeting ability are more efficient because they can rely on not only the EPR effect but the targeting moieties that can bind with specific receptors in the infection sites. Nanomedicines for targeting tumor microenvironments have been widely designed, and they have got favorable outcomes in tumor treatments (Kim et al., 2021). Different from tumor microenvironments, infectious microenvironments include both host cells and bacteria, and their formation is acute and temporary (Dong et al., 2019; Zhu et al., 2021). A lot of nanomedicines with active targeting ability to infectious microenvironments, to date, have been exploited to efficiently deliver antibiotics to infectious diseases.

Macrophages in infectious microenvironments are often targeted. Nanomedicines conjugating mannose enable active targeting to macrophages which express mannose receptors on the surface. Cai et al. have proved that photoacoustic agents can be modified with mannose to efficiently accumulate at infection sites (Cai et al., 2018a; Cai et al., 2018b). Besides, some antimicrobial peptides can be used as targeting moieties. A recent study reported by Yang et al. shows that gentamicin-loaded mesoporous silica nanoparticles can be labeled with a cationic human antimicrobial peptide fragment ubiquicidin (UBI)_29–41_ to target infection sites actively (Yang et al., 2018). However, the lack of corresponding receptors owing to the heterogeneity of infectious microenvironments may limit the efficiency of a single targeting factor.

**FIGURE 1:**
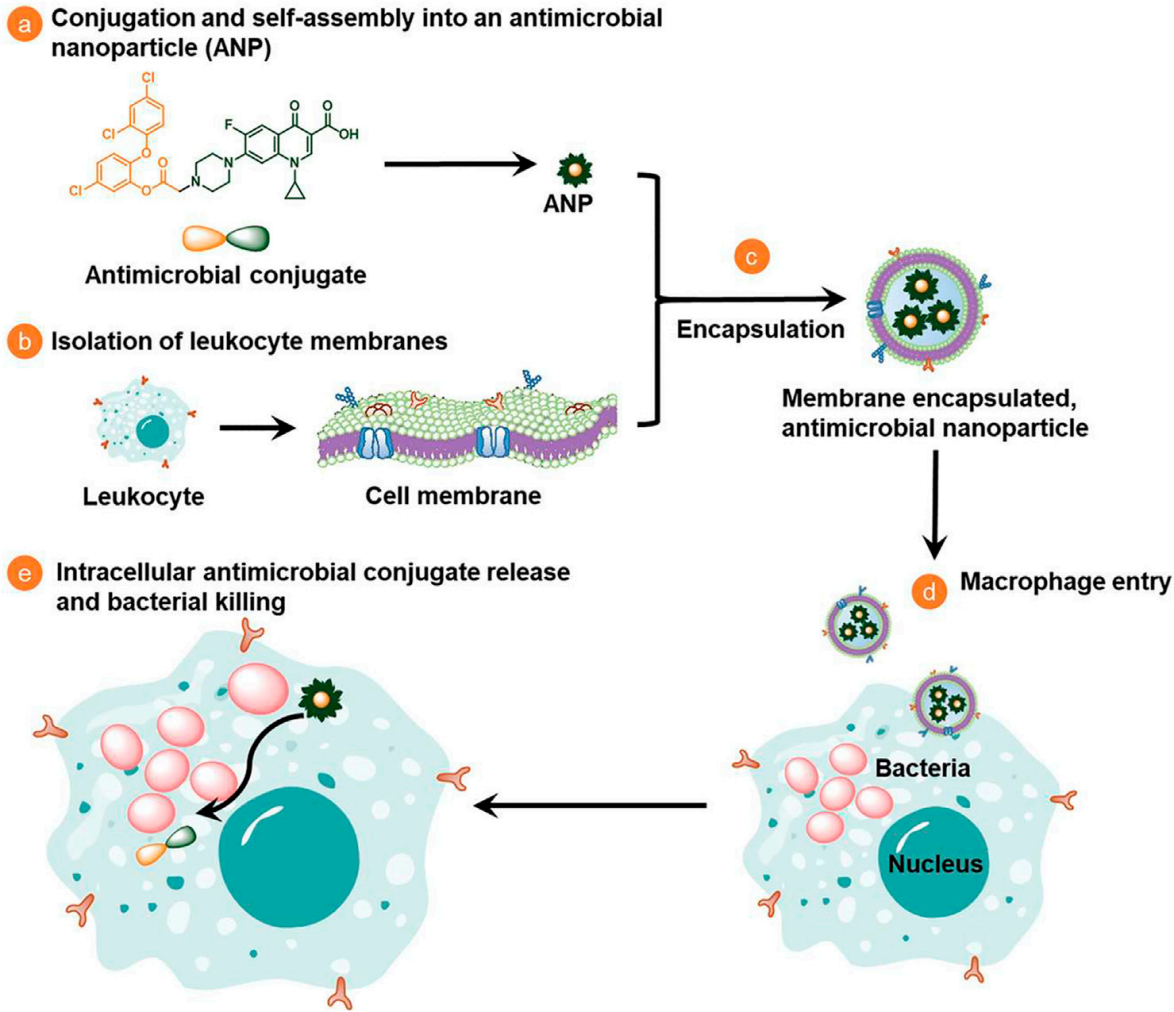
Schematic illustration on the design, synthesis, and potential mechanism of macrophage-monocyte membrane-encapsulated, antimicrobial-conjugated nanoparticles to kill intracellular bacteria (Li et al., 2020).

**FIGURE 2:**
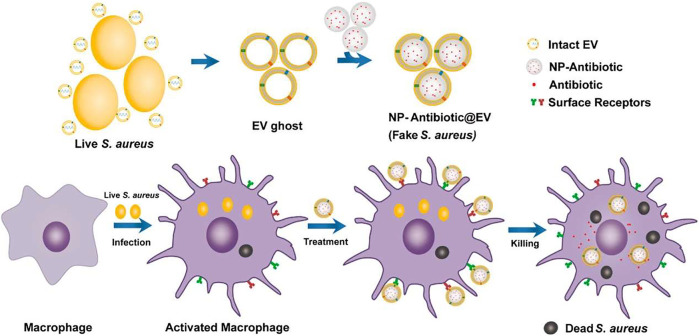
The preparation of nanomedicines by fusing the extracellular vesicles membrane derived from *S. aureus* (EV ghost) over the surface of a nanoparticle (NP) and their therapeutic process (Gao et al., 2019).

Biological membranes which contain a variety of ingredients have been applied to endow nanomedicines with multiple targeting capabilities to infection sites. As shown in Figure 1, Li et al. coat antimicrobial nanoparticles with macrophage membranes and these nanomedicines can be selectively taken up by infected macrophages/monocytes through the combination of surface Toll-like receptors and their electronegativity (Li et al., 2020). Extracellular vesicles have recently been used as drug delivery carriers to treat different diseases (Qi et al., 2021). Figure 2 shows that Gao et al. use nanoparticles that are coated with membranes of extracellular vesicles secreted by *Staphylococcus aureus* to deliver antibiotics, and these nanomedicines show significantly higher accumulations in kidney, lung, spleen, and heart bearing metastatic *Staphylococcus aureus* infections than in healthy counterparts (Gao et al., 2019). Furthermore, Bose et al. exploit a kind of apoptotic body-based active-targeting carriers. They use reconstructed apoptotic bodies derived from cancer cells to deliver vancomycin, and the resulting nanomedicines can take advantage of the inherent “eat me” signaling of apoptotic bodies to target infected organs (Bose et al., 2020). The versatility of biological membranes can potentially improve the targeting efficiency of nanomedicines, but the heterogeneity of biological membranes should be seriously considered. For example, membranes with different sizes and shapes may construct nanomedicines with distinct properties. Furthermore, the application of viruses that can target specific bacteria has been also used as a potential targeting approach, especially for pulmonary infections (Subramaniam et al., 2019).

It is worth noted that *in vitro* guidance can also realize the infection targeting of nanomedicines (Yu et al., 2020). As an example, magnetic targeting has been recently introduced to enhance the accumulation of nanomedicines at infection sites (Subbiahdoss et al., 2012; Armenia et al., 2018). Ji et al. utilize the magnetic targeting ability of magnetic nanoparticles to deliver glucose oxidase, efficiently eradicating bacterial/fungi biofilms (Ji et al., 2021). However, *in vitro* guidance is only suitable for detected infections. The combination of active targeting and *in vitro* guidance may further improve the targeting efficiency of nanomedicines.

## Recognition of Infected Cells

After accumulation at infection sites, nanomedicines should recognize the infected cells and interact with them, facilitating the delivery of antibiotics to intracellular bacteria. There are many types of cells at the site of infection, and recognizing different types of cells requires different methods.

Phagocytic cells, especially macrophages, are often responsible to engulf and remove bacteria. However, once the killing mechanisms of phagocytic cells are subverted, the engulfed bacteria can remain alive inside these cells (Imbuluzqueta et al., 2011). The recognition of these infected phagocytic cells is relatively easy because nanomedicines are readily captured by these cells similar to bacteria. For example, core-shell nanostructures encapsulating gentamicin based on commercial pluronic could be efficiently taken up by the tissue macrophages, resulting in a significant reduction of viable bacteria in the liver and spleen (Ranjan et al., 2009). Furthermore, nanomedicines can also be modified with phagocytic cell-targeting moieties as we have mentioned above to endow them with active targeting ability, further improving the uptake efficiency of phagocytic cells.

However, various intracellular bacteria, such as *Staphylococcus aureus* and *Salmonella*, can also locate in nonphagocytic cells including epithelial cells, fibroblasts, and hepatocytes. For example, *Mycobacterium tuberculosis* locates in both macrophages and hepatocytes (Barrios-Payán et al., 2012), and Listeria monocytogenes can persist in not only macrophages but hepatocytes and enterocytes (Barbuddhe and Chakraborty, 2009). To recognize and target these infected nonphagocytic cells, possible approaches are to increase the blood circulation time of nanomedicines by decreasing the uptake of phagocytic cells. Hyaluronan-based nanoparticles have high biocompatibility, so they can effectively accumulate in the skin to deliver antibiotics to dermis and epidermis cells such as keratinocytes and fibroblasts (Montanari et al., 2020; Zheng et al., 2021). Besides, hyaluronan-based nanoparticles also have active targeting ability because they can bind with CD44 isoforms which are highly expressed in dermis and epidermis cells. Therefore, coupling corresponding targeting moieties to nanomedicines can further improve its targeting efficiency to infected nonphagocytic cells. For example, *Mycobacterium* infected cells show increased uptake of arginine by the mediation of cationic transporters, and arginine-conjugated mesoporous silica nanoparticles can efficiently target *Salmonella* infected macrophages and epithelial cells (Mudakavi et al., 2017).

Recognition of infected cells, in fact, is often difficult. Infected cells do not show obvious differences from normal cells, and that is also the reason why bacteria in infected cells can escape from the immune system. To the best of our knowledge, there are no nanomedicines that can precisely recognize infected cells without targeting normal cells by far. Therefore, the recognition of infected cells needs new mechanisms as well as new methods.

## Targeting of Intracellular Bacteria

To date, various antibiotics with excellent *in vitro* activity often show limited *in vivo* therapeutic effects. One of the main reasons is that these antibiotics cannot reach the bacteria-harboring intracellular compartments leading to the low effective concentration (Hao et al., 2021). To enhance the therapeutic effects of antibiotics, therefore, nanomedicines need to target intracellular bacteria and release antibiotics to the bacteria-located intracellular compartments (He et al., 2021).

Bacteria may inhabit different intracellular compartments (Fenaroli et al., 2020). For example, Listeria monocytogenes would proliferate in the cytosol of cells, while Listeria pneumonia colonizes in the endoplasmic reticulum like vacuoles. *Salmonella enterica* often locates in the late endosomal compartments. Besides, the most studied intracellular bacteria, *Mycobacterium tuberculosis*, survives in phagosomes. Therefore, targeting these intracellular bacteria needs nanomedicines to locate in corresponding compartments.

In some cases, nanomedicines and bacteria would have the same pathway into cells. For these nanomedicines, they can inherently locate in the same compartments with intracellular bacteria, realizing the passive targeting effect. As an example, nanoparticles of mesoporous iron carboxylate metal-organic framework (nanoMOFs) can be internalized by macrophages, and the internalization pathway is principally phagocytosis. These nanoMOFs can colocalize with intracellular *Staphylococcus aureus* which locates in the phagosomes of macrophages and deliver two antibiotics to fight them (Li et al., 2019). Shi et al. show that iron oxide nanozymes co-localize with *Salmonella* Enteritidis in autophagic vacuoles, and they can promote the antibacterial effects of autophagic vacuoles by increasing reactive oxygen species levels (Shi et al., 2018). Furthermore, many nanomedicines mainly deliver antibiotics to the endosomes or cytoplasm, while bacteria could reside in different intracellular compartments such as vacuoles, nucleus, Golgi apparatus, and endoplasmic reticulum. These nanomedicines have to target intracellular bacteria by actively entering compartments in which bacteria are located, and they are often modified with targeting moieties (Wang et al., 2021; Yin et al., 2021). Yang et al. immobilize bacteria-targeting peptides on the surface shell of lipid nanoparticles to precisely deliver antibiotics, efficiently eliminating intracellular *Staphylococcus aureus* (Yang et al., 2018). Singh et al. conjugate sushi peptides which have a strong affinity for lipopolysaccharide (LPS) to gold nanoparticles effectively recognizing intracellular *Salmonella typhi* (Singh et al., 2017). Besides peptides, other targeting moieties can also be used. For example, Fu et al. report that aptamer-modified quantum dots can recognize intracellular *Staphylococcus aureus* (Fu et al., 2020). Furthermore, novel nanomedicines are exploited recently. Lehar et al. develop a kind of antibody-antibiotic conjugates to eliminate intracellular *Staphylococcus aureus*, and these antibody-antibiotic conjugates are superior to free antibiotics (Lehar et al., 2015).

## Conclusions and Future Perspective

The development of nanomedicines for bacterial infections has attracted wide attention, while the research on nanomedicines for intracellular bacteria is limited by far. Part of this is because nanomedicines need to overcome multiple physiological barriers to effectively target intracellular bacteria. They have to follow the “ART” principle, sequentially realizing the accumulation at the infection sites, recognization of the infected cells, and targeting the intracellular bacteria. For this purpose, therefore, they must be elaborately decorated according to the properties of targeted bacteria, raising the difficulty of designing nanomedicines. Endowing nanomedicines with the targeting ability to the infection sites is a relatively easy step because there would be obvious abnormalities at the infection sites such as vascular heterogeneity and abnormally high expression of certain receptors. By contrast, the precise recognization of the infected cells is still an arduous challenge. The differences between the infected cells and normal cells are often negligible, and the immune system sometimes fails to detect these differences. The specific cellular markers of the infected cells should be screened by the latest biological tests including proteome analysis and sequencing analysis, facilitating the development of targeting moieties. Furthermore, the targeting of intracellular bacteria often needs nanomedicines to possess the ability to respond to the environment. On the one hand, nanomedicines should escape from their intracellular compartments to bacteria-located compartments through their responsiveness. On the other hand, nanomedicines should responsively release antibiotics to bacteria-located compartments. Therefore, nanomedicines should have versatility including active targeting ability and environmental responsiveness for the follow of the “ART” principle.

The other main reason for limited researches on nanomedicines using to eliminate intracellular bacteria is that the construction methods of experimental models are not perfect. In current researches, cells are often co-cultured with bacteria to simulate the model of intracellular bacterial infection. During this process, bacteria may induce the death of cells, while intracellular bacteria are often symbiotic with cells in reality. Under the circumstances, nanomedicines would face a situation that is completely different from reality. Furthermore, the same is true of the construction of animal models. There is no definitive way to prove that bacteria directly applied to animals are certainly located in the intracellular compartments. We have noted that the construction of the intracellular bacteria-infected animal models is similar to that of routine bacteria-infected animal models in current studies, potentially leading to the false-positive therapy effects of nanomedicines on intracellular bacteria-induced infections. Therefore, the researches on nanomedicines for intracellular bacteria require the golden approach for the building of cell and animal experimental models. In addition, other kinds of drugs should also be exploited, such as nucleic acid drugs (Li et al., 2021; Yang et al., 2021; Zong et al., 2021), for the clearance of intracellular bacteria.

In conclusion, it has been harder to develop new antibiotics recently, and nanomedicines have emerged as one of the optimal choices for infections induced by intracellular bacteria. To further realize the clinical translation of these nanomedicines, however, the golden approach for the building of cell and animal experimental models should be established. Furthermore, nanomedicines should be also endowed with the versatility to follow the “ART” principle. For this purpose, they should be modified with targeting moieties that could target infection sites, infected cells, and intracellular bacteria respectively.
